# Clinical outcomes after implantation of a new monofocal intraocular lens with enhanced intermediate function in patients with preperimetric glaucoma

**DOI:** 10.3389/fmed.2023.1260298

**Published:** 2024-01-04

**Authors:** Ho Seok Chung, Joon Hyuck Jang, Hun Lee, Jae Yong Kim, Hungwon Tchah

**Affiliations:** ^1^Department of Ophthalmology, Asan Medical Center, University of Ulsan College of Medicine, Seoul, Republic of Korea; ^2^Department of Ophthalmology, Kim's Eye Hospital, Myung-Gok Eye Research Institute, Konyang University College of Medicine, Seoul, Republic of Korea

**Keywords:** monofocal intraocular lenses with enhanced intermediate function, retinal nerve fiber layer defect, cataract surgery, preperimetric glaucoma, cataract

## Abstract

**Purpose:**

We evaluated the clinical outcomes after implantation of a new monofocal intraocular lens (IOL) with enhanced intermediate function in patients with preperimetric glaucoma and compared those with patients without retinal nerve fiber layer (RNFL) defects.

**Methods:**

All patients were implanted bilaterally a new monofocal IOL with enhanced intermediate function. Patients with bilateral RNFL defects and no glaucomatous visual field defects were classified as the preperimetric glaucoma group. A total of 60 eyes of 30 patients with preperimetric glaucoma were compared with 60 eyes of 30 patients without RNFL defects. Uncorrected distance visual acuity (UDVA), uncorrected intermediate visual acuity (UIVA), uncorrected near visual acuity (UNVA), defocus curve, contrast sensitivity, and questionnaire were evaluated 1 month and 3 months after surgery.

**Results:**

No difference in binocular UDVA, UIVA, and UNVA was evident between the two groups at 1 and 3 months postoperatively. Additionally, there were no significant differences between the two groups regarding the proportion of severe or very severe photic phenomena, such as glare and halos, or the overall satisfaction.

**Conclusion:**

Bilateral implantation of a new monofocal IOL with enhanced intermediate function in patients with preperimetric glaucoma demonstrated commensurate clinical outcomes and could be considered a feasible alternative.

## Introduction

Cataract surgery is one of the most common surgeries performed worldwide. Recently, a variety of multifocal intraocular lenses (IOLs) were introduced. These are widely implanted during cataract surgery to allow for near visual acuity without spectacles. Compared to monofocal IOLs, multifocal IOLs demonstrated better uncorrected near visual acuity (UNVA), which made patients spectacle-free (1–6). Owing to an increase in the usage of smartphones, tablets, and personal computers, it is becoming more important to maintain near to intermediate visual acuity. However, it is also crucial to prevent adverse events associated with multifocal IOLs (4, 7, 8).

In real-world practice, photic phenomena such as halo and glare and reduced contrast sensitivity in low-light conditions influence patient’s visual quality (1, 7, 9). Conventional monofocal IOLs demonstrated good contrast sensitivity and low incidence of photic phenomena (6, 10–12). A new monofocal IOL with enhanced intermediate function (ICB00, tecnis eyhance, Johnson & Johnson Vision Care, Inc. Santa Ana, CA, USA) extends focus in the longitudinal plane. Extended focus prevents the overlap of near and far images, minimizing the halo effect. This IOL is based on the continuous power change from periphery to center of the anterior surface without a demarcation line (5). Theoretically, this unique anterior structure provides better distance and intermediate vision than conventional monofocal IOLs in addition to fewer photic effects than those by diffractive multifocal IOLs (6, 11). The ICB00 demonstrated comparable uncorrected distance visual acuity (UDVA) and better uncorrected intermediate visual acuity (UIVA) compared to that of aspheric monofocal IOL (ZCB00, Tecnis, Johnson & Johnson Vision Care, Inc), although UNVA was low in both IOLs. Additionally, with respect to contrast sensitivity and photic phenomena, previous studies have demonstrated good visual quality in patients with the implantation of the ICB00 (5, 13).

However, the clinical outcome with the implantation of the ICB00 in real-world circumstances is still unclear, particularly for patients with preperimetric glaucoma. Theoretically, the extended depth-of-focus technique does not influence contrast sensitivity (6). However, some patients with ophthalmic problems, such as preperimetric glaucoma, already have low baseline contrast sensitivity. In such cases, post-operative visual quality and contrast sensitivity could be influenced by IOL design. Therefore, in the present study, we aimed to evaluate clinical outcomes of the new monofocal IOL with enhanced intermediate function in patients with preperimetric glaucoma by comparing those in patients without retinal nerve fiber layer (RNFL) defects.

## Methods

### Participants

The study followed the principles of the Declaration of Helsinki and was approved by the Institutional Review Board of Asan Medical Center (2022–0365). Written informed consent was obtained from each participant after explaining the purpose of the study. As a non-randomized prospective comparative study, this study recruited patients who underwent binocular cataract surgery at the Department of Ophthalmology, Asan Medical Center, Seoul, South Korea between January 2020 and January 2022. The RNFL thickness of average and quadrant value was measured by cirrus spectral-domain optical coherence tomography (Carl Zeiss Meditec, Inc., Dublin, CA, USA). All patients underwent the same cataract surgery, with same IOLs in both eyes (ICB00). RNFL defect was defined when RNFL thickness was below 1% of normal distribution in at least one quadrant in both eyes. Patients were classified into two groups based on RNFL thickness measurements. Patients with bilateral RNFL defects and no glaucomatous visual field defect in both eyes were classified as the preperimetric glaucoma group, and patients without bilateral RNFL defects were classified as the control group. Furthermore, patients who had potential visual acuity over 20/25 of Snellen visual acuity in corrected distance visual acuity (CDVA) in both eyes and preoperative corneal astigmatism of ≤1.50 diopters (D) in both eyes were included. Patients were excluded based on the following criteria: (i) previous ocular trauma, (ii) previous ocular surgery including refractive surgery, (iii) corneal irregularities or abnormalities including corneal opacities, (iv) systemic or ocular medication that could influence vision, and (v) glaucomatous visual field defects corresponding to RNFL defects in either eye.

### New monofocal IOL with enhanced intermediate function

Eyhance IOL is a new monofocal IOL characterized by a continuous power change from the periphery to the center of the IOL (14). It can provide comparable distant visual acuity with monofocal IOL and could improve the intermediate visual performance while minimizing the photic phenomena such as glare and halo (5). Eyhance IOL has the same appearance as an aspheric monofocal IOL (ZCB00) from the same manufacturer. It has a biconvex shape with continuous high-order aspheric anterior surface and spherical posterior surface. It provides a negative spherical aberration of-0.27 μm (14).

### Surgical techniques

Femtosecond laser Catalyst FSL platform (Johnson & Johnson Vision Care, Inc) was used for all patients. The standard procedure was carried out as follows. Treatment was initiated using suction ring and applanation cone. Continuous curvilinear capsulorhexis and lens fragmentation were performed, followed by arcuate keratotomy. Intrastromal arcuate keratotomy was performed based on nomograms in the device. The programmed anterior capsulotomy was 4.8 mm in diameter. Crystalline lens fragmentation was done using a standard template with a pattern described as “lens softening: quadrants” in the system. A clear corneal incision of 2.2 mm was made. Hydrodissection and phacoemulsification were performed. A new monofocal IOL with enhanced intermediate function (ICB00, tecnis eyhance) was then implanted into the capsular bag using an injector, and all incisions were hydrosealed without sutures. Same surgical procedure was performed on the fellow eye.

### Preoperative and postoperative examinations

Preoperative corneal astigmatism was measured using an auto-keratometry device (Canon R-50, Canon USA Inc., Huntington, NY, USA), and axial length was measured using IOL master 700 (Carl Zeiss Meditec, Inc.). Preoperative spherical equivalent from manifest refraction, UDVA, and CDVA were measured. Binocular UDVA, CDVA, UIVA, and UNVA were measured at 1 and 3 months postoperatively. At postoperative 3 months, the binocular defocus curve was generated from +0.5 D to-4 D. Contrast sensitivity was measured at distance level in each uncorrected eye under photopic (85 cd/m^2^) and mesopic (3 cd/m^2^) conditions using the Functional Acuity Contrast Test function of the Ophtec 6,500 view-in test system (Stereo Optical Co, Inc., Chicago, IL, USA). Contrast sensitivity was determined at multiple spatial frequencies ranging from 1.5 to 18 cycles per degree (cpd) (1.5, 3, 6, 12, and 18 cpd). A questionnaire about overall satisfaction, visual symptoms, spectacle dependence for near vision, and willingness to recommend IOL to others was conducted after surgery. Overall patient satisfaction was determined using a 5-point Likert scale, with 1 = very dissatisfied, 2 = dissatisfied, 3 = neither satisfied nor dissatisfied, 4 = satisfied, and 5 = very satisfied. Photic phenomena (glare and halo) were evaluated on a 5-point scale from 1 (no symptoms) to 5 (very severe symptoms). Spectacle dependence was evaluated on a 5-point scale from 1 (always) to 5 (never). Additionally patients answered whether they would recommend implantation of IOLs to their friends or relatives, with “yes” or “no” as responses.

### Statistical analysis

All statistical analyses were performed using IBM SPSS Statistics V21.0 (IBM Corp., Armonk, New York, USA). The independent t-test for unpaired data was used for comparison between the two groups. When parametric analysis was unavailable, the Mann–Whitney U test was used. Categorical variables were analyzed using the chi-square test. *p* values of less than 0.05 were considered statistically significant.

## Results

This study included 120 eyes of 60 patients (preperimetric glaucoma group: 30 patients, control group: 30 patients). There were no differences in preoperative clinical characteristics between the two groups except for RNFL thickness. RNFL thickness was thinner on average and in all quadrants in the preperimetric glaucoma group than in the control group (Table 1).

**Table 1:** Comparison of the preoperative clinical characteristics between the preperimetric glaucoma and control groups.

Parameter	Preperimetric glaucoma group (30 patients)	Control group (30 patients)	*p* value
Age (year)	72.73 (6.34)	70.42 (5.63)	0.197
Sex (male:female)	16:14	12:18	0.527
Preoperative UDVA (logMAR)	0.43 (0.23)	0.33 (0.23)	0.105
Preoperative CDVA (logMAR)	0.29 (0.25)	0.33 (0.22)	0.414
Spherical equivalent (D)	−2.02 (3.87)	−1.67 (2.66)	0.626
Corneal astigmatism (D)	0.81 (0.41)	0.81 (0.54)	0.973
Axial length (mm)	24.11 (2.34)	24.24 (1.35)	0.741
RNFL thickness			
Superior (μm)	89.48 (23.58)	118.02 (14.37)	<0.001
Inferior (μm)	85.16 (24.33)	118.10 (27.14)	<0.001
Nasal (μm)	64.41 (20.67)	80.63 (24.33)	<0.001
Temporal (μm)	63.00 (18.66)	87.23 (23.33)	<0.001
Average (μm)	76.60 (15.47)	96.93 (11.80)	<0.001

Data are presented as a mean value (SD).

UDVA, uncorrected distant visual acuity; CDVA, corrected distant visual acuity; D, diopter; RNFL, retinal nerve fiber layer; logMAR, logarithm of the minimum angle of resolution.

**Figure 1:**
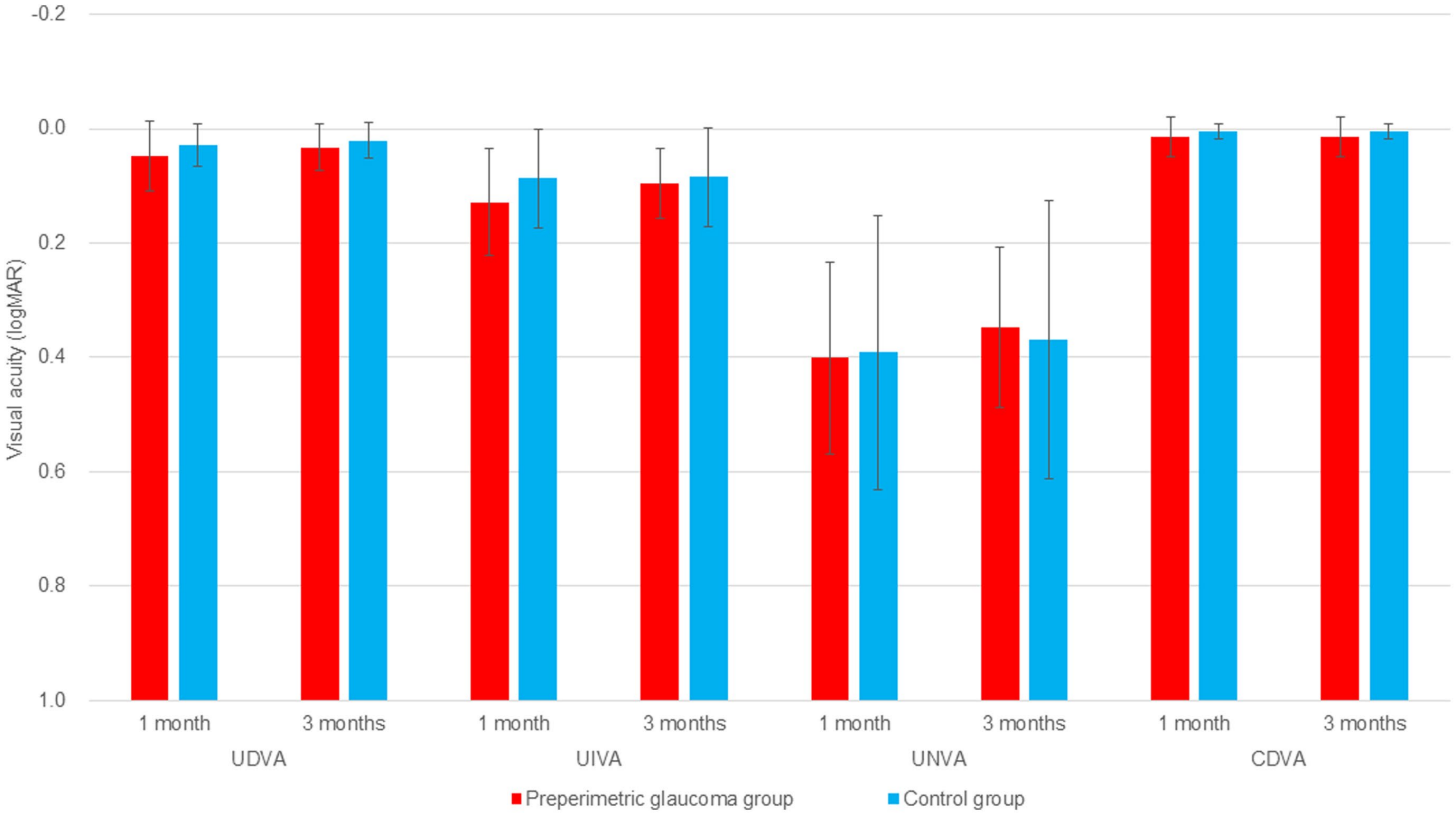
Comparison of 1 and 3 months postoperative distance, intermediate, and near visual acuities in the preperimetric glaucoma and control groups. logMAR, logarithm of the minimum angle of resolution; RNFL, retinal nerve fiber layer; UDVA, uncorrected distance visual acuity; UIVA, uncorrected intermediate visual acuity; UNVA, uncorrected near visual acuity; CDVA, corrected distance visual acuity.

**Figure 2:**
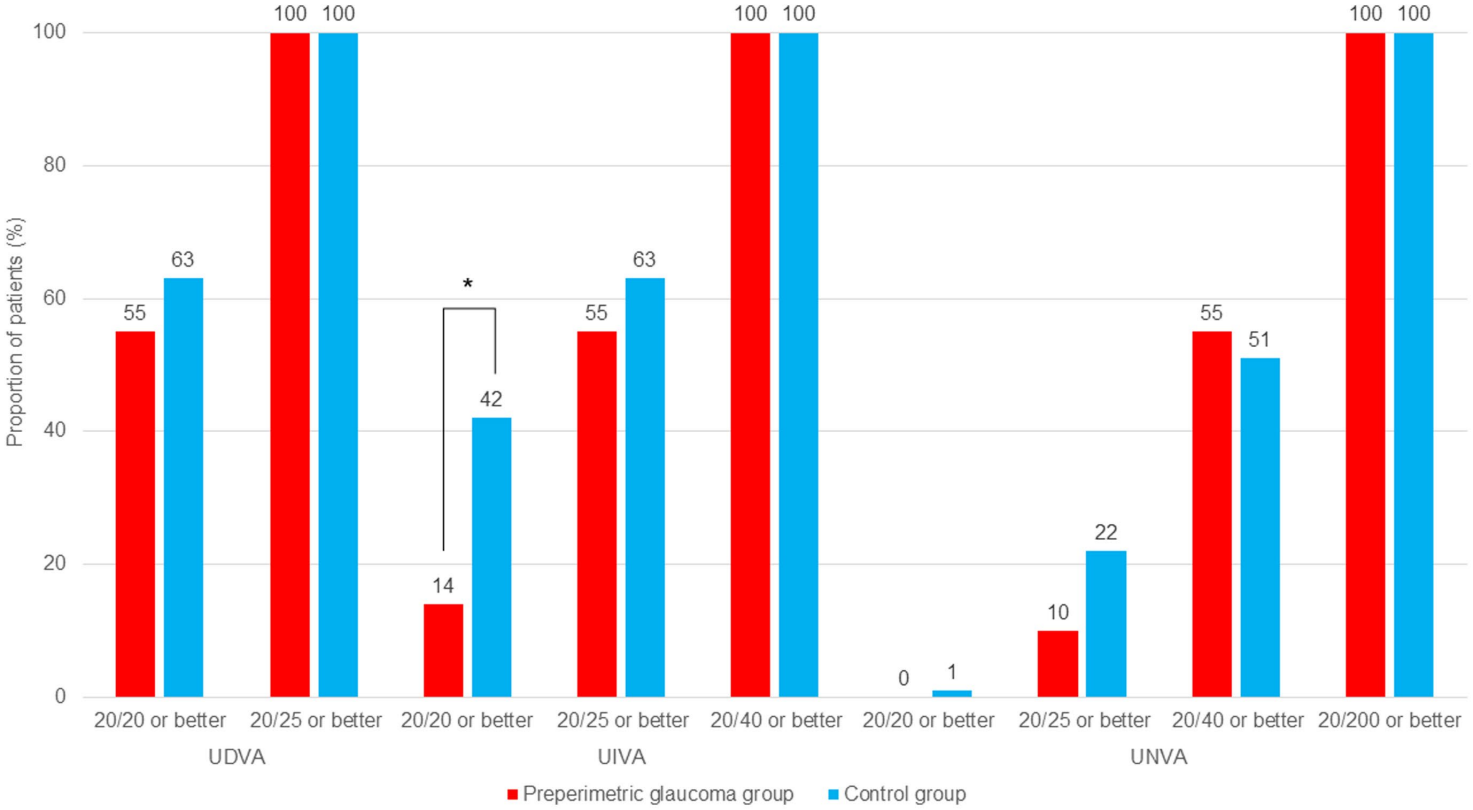
Comparison of cumulative distribution of uncorrected binocular visual acuities at postoperative 3 months in the preperimetric glaucoma and control groups. RNFL, retinal nerve fiber layer; UDVA, uncorrected distance visual acuity; UIVA, uncorrected intermediate visual acuity; UNVA, uncorrected near visual acuity. *p < 0.05 between groups using chi-square test.

At postoperative 1 and 3 months, binocular CDVA was not statistically different between the preperimetric glaucoma and control groups (*p* = 0.148 for 1 month, and *p* = 0.137 for 3 months). Binocular UDVA, UIVA, and UNVA were not different between the two groups at 1 and 3 months postoperatively (Table 2; Figure 1). Regarding the UDVA, the proportion of patients with 20/20 or better was similar between the preperimetric glaucoma and control groups (55% for former versus 63% for latter, *p* = 0.765) (Figure 2). Regarding the UIVA, the proportion of patients with 20/25 or better was similar between the preperimetric glaucoma and control groups (55% versus 63%, p = 0.765). However, the proportion of patients with 20/20 or better was higher in the control group thanin the preperimetric glaucoma group (42% for former versus 14% for latter, *p* = 0.040) (Figure 2). Regarding the UNVA, the proportion of patients with 20/40 or better was similar between the two groups (Figure 2).

**Table 2:** Comparison of the 1-and 3-month postoperative binocular visual acuities between the preperimetric glaucoma and control groups.

Visual acuity (LogMAR)		Preperimetric glaucoma group (30 patients)	Control group (30 patients)	*p* value
Postoperative UDVA	1 M	0.05 (0.06)	0.03 (0.06)	0.187
	3 M	0.03 (0.04)	0.02 (0.03)	0.305
Postoperative UIVA	1 M	0.13 (0.09)	0.09 (0.09)	0.125
	3 M	0.10 (0.06)	0.09 (0.09)	0.622
Postoperative UNVA	1 M	0.40 (0.39)	0.39 (0.17)	0.879
	3 M	0.35 (0.14)	0.37 (0.24)	0.707
Postoperative CDVA	1 M	0.02 (0.04)	0.01 (0.01)	0.148
	3 M	0.02 (0.03)	0.004 (0.01)	0.137

Data are presented as a mean value (SD).

RNFL, retinal nerve fiber layer; CDVA, corrected distant visual acuity; UDVA, uncorrected distant visual acuity; UIVA, uncorrected intermediate visual acuity; UNVA, uncorrected near visual acuity; logMAR, logarithm of the minimum angle of resolution.

**Figure 3:**
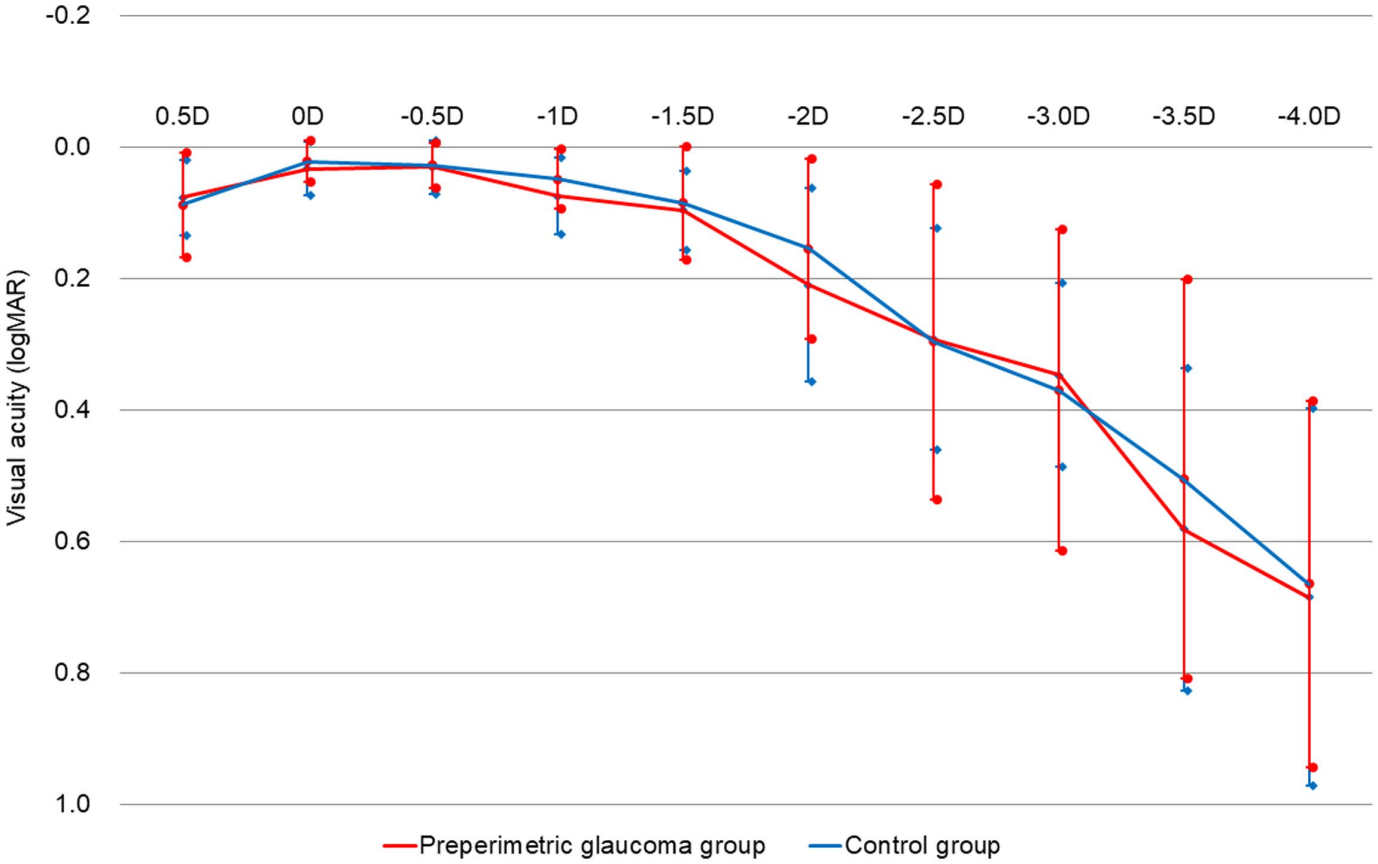
Postoperative 3-month binocular defocus curves of the preperimetric glaucoma and control groups. D; diopters; logMAR, logarithm of the minimum angle of resolution; RNFL, retinal nerve fiber layer.

Figure 3 shows the binocular defocus curve measured in both groups. Binocular visual acuity is highest in defocus 0.00 D, and as defocus becomes negative, visual acuity slowly decreases. In most defocus indices, visual acuity was not statistically different between the two groups. Although not significant statistically, defocus curves of the two groups showed the tendency that the overall visual acuity of the preperimetric glaucoma group was lower than that of the control group.

**Figure 4:**
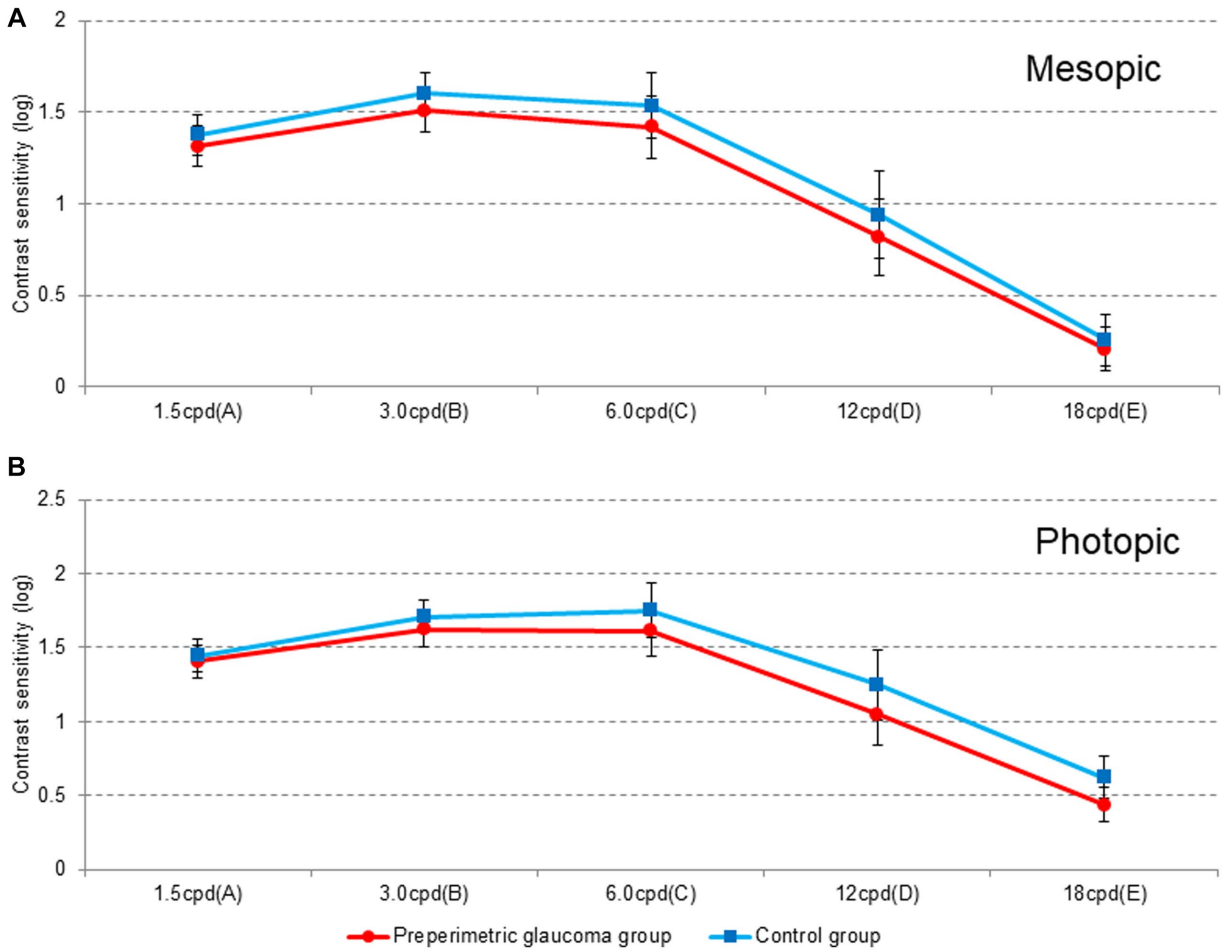
Contrast sensitivity under **(A)** mesopic and **(B)** photopic conditions at different spatial frequencies (cycles per degree) at 3 months postoperatively. Cpd, cycles per degree; RNFL, retinal nerve fiber layer.

Figure 4 shows contrast sensitivity results in photopic and mesopic conditions. In all spatial frequencies and both photopic and mesopic conditions, there was no significant difference in contrast sensitivity between the two groups (Figures 4A,B). The control group demonstrated better contrast sensitivity results, albeit not significant statistically.

**Figure 5:**
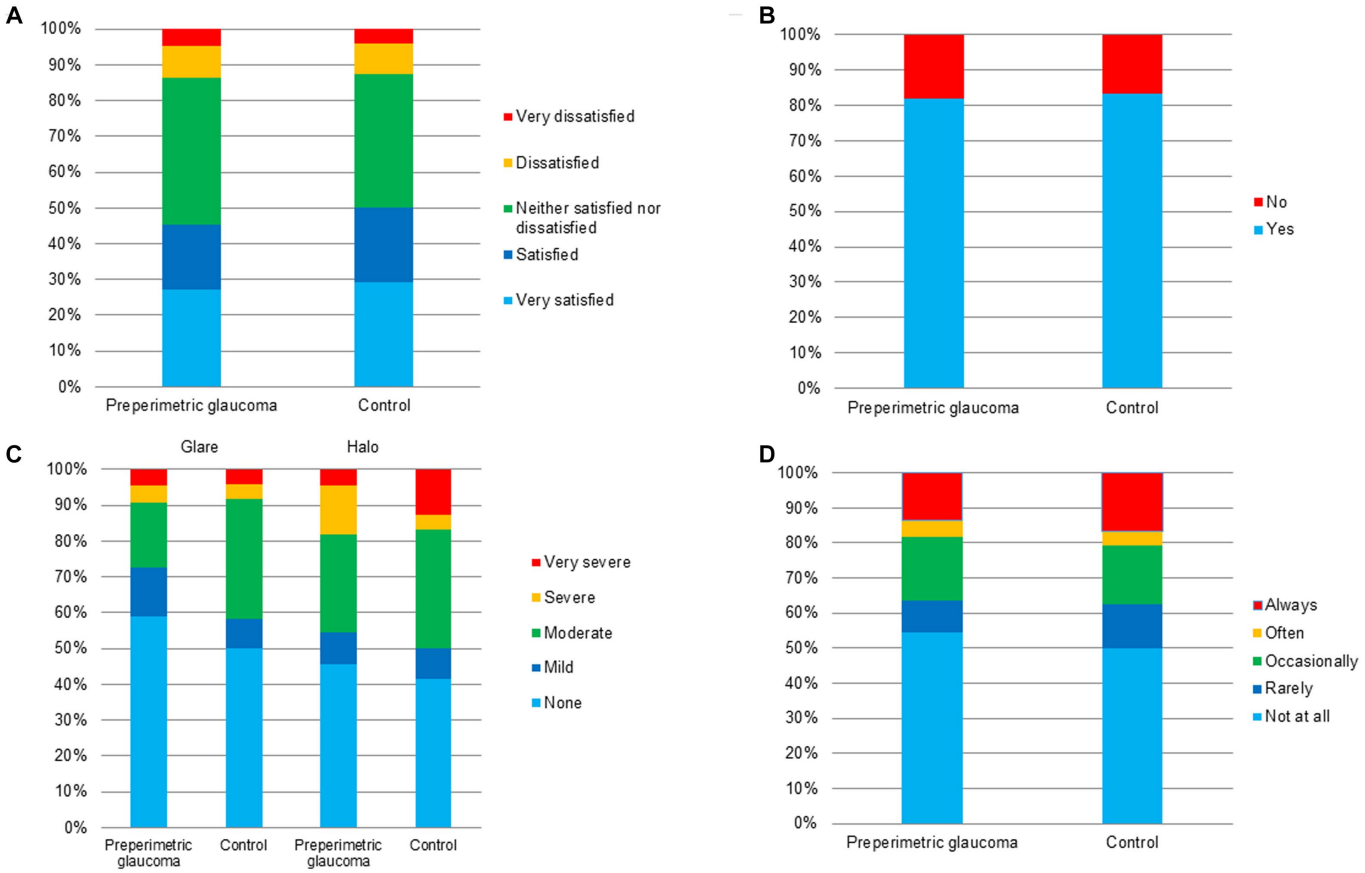
Questionnaire results of the preperimetric glaucoma and control groups. **(A)** Overall satisfaction, **(B)** recommendation to others, **(C)** photic phenomena including glare and halo, and **(D)** spectacle independence.

In terms of the overall subjective satisfaction, the proportion of very satisfied, satisfied, and neither satisfied nor dissatisfied patients was 86.7% (26/30) and 83.3% (25/30) in the preperimetric glaucoma and control groups, respectively (Table 3; Figure 5A). The proportion of those who would recommend the surgery to others was 83.3% (25/30) in the preperimetric glaucoma group and 83.3% (25/30) in the control group (Table 3; Figure 5B). Regarding the photic phenomena, the proportion of glare (severe or very severe) was 10.0% (3/30) in the preperimetric glaucoma group and 10.0% (3/30) in the control group (Table 3; Figure 5C). The proportion of halo (severe or very severe) was 20.0% (6/30) in the preperimetric glaucoma group and 16.7% (5/30) in the control group (Table 3; Figure 5C). The proportion of spectacle dependence (always) at near vision was 13.3% (4/30) in the preperimetric glaucoma group and 16.7% (5/30) in the control group (Table 3; Figure 5D).

**Table 3:** Subjective satisfaction, spectacle usage, glare, halo, and recommendation between the preperimetric glaucoma and control groups.

Characteristics	Preperimetric glaucoma group (30 patients)	Control group (30 patients)
Overall satisfaction 3–5 1–2	26 (86.7%) 4 (13.3%)	25 (83.3%) 5 (16.7%)
Recommendation Yes No	25 (83.3%) 5 (16.7%)	25 (83.3%) 5 (16.7%)
Glare No/mild glare Moderate glare Severe/Very severe glare	22 (73.3%) 5 (16.7%) 3 (10.0%)	17 (56.7%) 10 (33.3%) 3 (10.0%)
Halo No/mild halo Moderate halo Severe/Very severe halo	16 (53.3%) 8 (26.7%) 6 (20.0%)	15 (50.0%) 10 (33.3%) 5 (16.7%)
Spectacle usage No usage Always	16 (53.3%) 4 (13.3%)	15 (50.0%) 5 (16.7%)

Overall satisfaction: 1 = very dissatisfied, 2 = dissatisfied, 3 = neither satisfied nor dissatisfied, 4 = satisfied, and 5 = very satisfied.

## Discussion

In the present study, patients with preperimetric glaucoma with cataract extraction and implantation of the ICB00 showed decreased visual acuity and contrast sensitivity when compared with patients without any RNFL defects receiving the same surgery with the same IOL. However, decrease in visual acuity and contrast sensitivity was not significantly prominent. Moreover, there was no significant difference in patients’ subjective satisfaction and photic phenomena scores between the two groups. Based on the results of our study, we suggest that bilateral implantation of a new monofocal IOL with enhanced intermediate function can be considered a good alternative in patients with preperimetric glaucoma considering cataract extraction and IOL implantation.

Glaucoma is one of the most common ocular diseases that decrease contrast sensitivity. In early and advanced glaucoma, contrast sensitivity decreases in all spatial frequencies (low, medium, and high) under both photopic and mesopic conditions (15–20). As the implantation of multifocal IOLs also causes decreased contrast sensitivity, multifocal IOL implantation in patients with moderate to severe glaucoma is relatively contraindicated (3, 5, 21, 22). Although a reported contraindication exists, a recent study demonstrated the subjective and objective outcomes after implantation of diffractive multifocal IOLs in patients with glaucoma and preperimetric glaucoma (23). In this study, there was no significant difference in visual outcomes between patients with preperimetric glaucoma and healthy controls; however, patients with glaucoma showed significant visual acuity loss at most distances and decreased contrast sensitivity. Another case series also demonstrated the degradation of visual function after extended depth-of-focus IOL implantation in patients with normal tension glaucoma without central visual field defects (24). Of 15 eyes, four showed lower contrast sensitivity in high spatial frequency than in the normal range. Therefore, multifocal or extended depth-of-focus IOLs should be implanted with caution in selected patients with glaucoma, considering the possibility of patient dissatisfaction due to decreased visual outcomes and contrast sensitivity.

In our study, patients with bilateral RNFL defects and no glaucomatous visual field defects in both eyes were included, and RNFL defect was defined as an RNFL thickness below 1% of normal distribution at least in one quadrant in both eyes. In general, a new monofocal IOL with enhanced intermediate function (ICB00) showed no significant difference in contrast sensitivity compared with standard monofocal IOLs using the same platform (ZCB00, Johnson & Johnson Vision Care, Inc. Santa Ana, CA, USA) (25–27). Given the nature of ICB00 and inclusion of patients with preperimetric glaucoma alone, contrast sensitivity in the preperimetric glaucoma group was not significantly different from that in the control group. Furthermore, a comparative study of ICB00 implantation with extended depth-of-focus IOL implantation in patients with preperimetric glaucoma or a study on the clinical outcomes of ICB00 implantation in patients with different degrees of glaucoma is required to verify and augment the results of the current study.

Postoperative distance, intermediate, and near visual acuities were not statistically different between the two groups at postoperative 1 and 3 months, as well as the defocus curve at postoperative 3 months. Although average UIVA was not different between the two groups at postoperative 1 and 3 months, the proportion of patients with 20/20 or better UIVA was significantly higher in the control group than in the preperimetric glaucoma group at 3 months postoperatively. Regarding the comparison of visual acuity at 1 and 3 months postoperatively, both groups demonstrated better visual acuity at 3 months than that at 1 month. The current results of better distance, intermediate, and near visual acuities at 3 months postoperatively, regardless of the presence of RNFL defects, can be attributed to individual adaptation to the new monofocal IOL with enhanced intermediate function, such as neural adaptation.

To accurately assess the impact of cataract surgery involving the implantation of a new monofocal IOL with enhanced intermediate function on contrast sensitivity compared to the aspheric monofocal IOL (ZCB00), the mean difference before and after cataract surgery using ICB00 needs to be compared with the results obtained by using ZCB00. However, cataract itself results in reduced contrast sensitivity; therefore, direct comparison of contrast sensitivity before and after surgery is not accurate. Thus, we evaluated the contrast sensitivity at 3 months postoperatively under photopic (85 cd/m^2^) and mesopic (3 cd/m^2^) conditions. In our study, although a minor trend of lower contrast sensitivity than that of the control group was observed in the preperimetric glaucoma group, the difference between the two groups was not statistically significant. Based on the current results, no difference in patients’ subjective satisfaction or postoperative visual acuity was observed between the two groups; therefore, we suspected that the minor difference in contrast sensitivity did not affect postoperative overall satisfaction and visual acuity.

To investigate the subjective outcomes of bilateral implantation of the new monofocal IOL with enhanced intermediate function, we conducted a survey using the questionnaire for subjective satisfaction, spectacle usage, glare, halo, and recommendation to others. Regarding the photic phenomena, there was no significant difference in the proportion of glare and halo (severe or very severe) between the two groups. In the present study, only three patients (10%) complained of severe/very severe glare in both groups, and six (20%) and five (16.7%) patients complained of severe/very severe halo in the preperimetric glaucoma and control groups, respectively. These results were in line with those of previous studies (12, 27). Regarding overall satisfaction, there was no significant difference in the proportion of very satisfied, satisfied, and neither satisfied nor dissatisfied patients between the two groups. These results showed non-inferiority when compared to those of previous studies that investigated the effect of implantation of a new monofocal IOL with enhanced intermediate function during cataract surgery (12, 13, 27, 28).

We analyzed clinical outcomes of patients with preperimetric glaucoma and found non-inferior subjective and objective results compared with the control group. Consistent with the findings of previous studies, implantation of a new monofocal IOL with enhanced intermediate function in our study demonstrated good distance and intermediate visual acuities and overall satisfaction in both groups (12). Although a new monofocal IOL with enhanced intermediate function is not diffractive multifocal IOL, the proportion of patients who did not require spectacles for near vision were 53.3 and 50.0% in the preperimetric glaucoma and control groups, respectively. Compared to multifocal IOLs, monofocal IOLs have an advantage of better contrast sensitivity and less photic phenomena, but lower near visual acuity and less spectacle-independence (7, 8, 12, 13, 27). Therefore, instead of multifocal IOLs which decrease contrast sensitivity and increase photic phenomena, a new monofocal IOL with enhanced intermediate function implantation could be an effective alternative for patients with preperimetric glaucoma seeking improved postoperative visual acuity without any severe glare or halo.

Limitations of the present study include the small sample size and a relatively short follow-up period. Further studies with a larger sample size and longer follow-up period are necessary to validate the results of the present study. Additionally, our results could be expanded by investigating clinical findings, such as visual field changes and contrast sensitivity, after implantation of the monofocal IOL with enhanced intermediate function in patients with glaucomatous optic disc changes and visual field defects.

In summary, bilateral cataract extraction and implantation of a new monofocal IOL with enhanced intermediate function in patients with preperimetric glaucoma demonstrated good subjective and objective outcomes and could be considered as a feasible alternative in patients with preperimetric glaucoma considering cataract extraction and IOL implantation.

## Data availability statement

The raw data supporting the conclusions of this article will be made available by the authors, without undue reservation.

## Ethics statement

The studies involving humans were approved by the study followed the principles of the Declaration of Helsinki and was approved by the Institutional Review Board of Asan Medical Center (2022-0365). The studies were conducted in accordance with the local legislation and institutional requirements. The participants provided their written informed consent to participate in this study. Written informed consent was obtained from the individual(s) for the publication of any potentially identifiable images or data included in this article.

## Author contributions

HC: Conceptualization, Data curation, Formal analysis, Investigation, Methodology, Visualization, Writing – original draft, Writing – review & editing. JJ: Conceptualization, Data curation, Formal analysis, Investigation, Methodology, Writing – original draft. HL: Conceptualization, Data curation, Formal analysis, Funding acquisition, Investigation, Supervision, Writing – original draft, Writing – review & editing. JK: Conceptualization, Data curation, Formal analysis, Investigation, Methodology, Writing – review & editing. HT: Conceptualization, Data curation, Formal analysis, Investigation, Methodology, Project administration, Supervision, Writing – review & editing.
